# Radiation-induced senescence: therapeutic opportunities

**DOI:** 10.1186/s13014-022-02184-2

**Published:** 2023-01-13

**Authors:** Jae Ho Kim, Stephen L. Brown, Marcia N. Gordon

**Affiliations:** 1 Radiobiology Research Laboratories, Department of Radiation Oncology, Henry Ford Health, 2799 West Grand Boulevard, Detroit, MI 48202 USA; 2 Department of Translational Neuroscience, Michigan State University, Grand Rapids, MI 49503 USA

**Keywords:** Radiation injuries, Cellular senescence, Senescence-associated secretory phenotype, Senotherapeutics

## Abstract

The limitation of cancer radiotherapy does not derive from an inability to ablate tumor, but rather to do so without excessively damaging critical tissues and organs and adversely affecting patient’s quality of life. Although cellular senescence is a normal consequence of aging, there is increasing evidence showing that the radiation-induced senescence in both tumor and adjacent normal tissues contributes to tumor recurrence, metastasis, and resistance to therapy, while chronic senescent cells in the normal tissue and organ are a source of many late damaging effects. In this review, we discuss how to identify cellular senescence using various bio-markers and the role of the so-called senescence-associated secretory phenotype characteristics on the pathogenesis of the radiation-induced late effects. We also discuss therapeutic options to eliminate cellular senescence using either senolytics and/or senostatics. Finally, a discussion of cellular reprogramming is presented, another promising avenue to improve the therapeutic gain of radiotherapy.

## Introduction

**Fig. 1:**
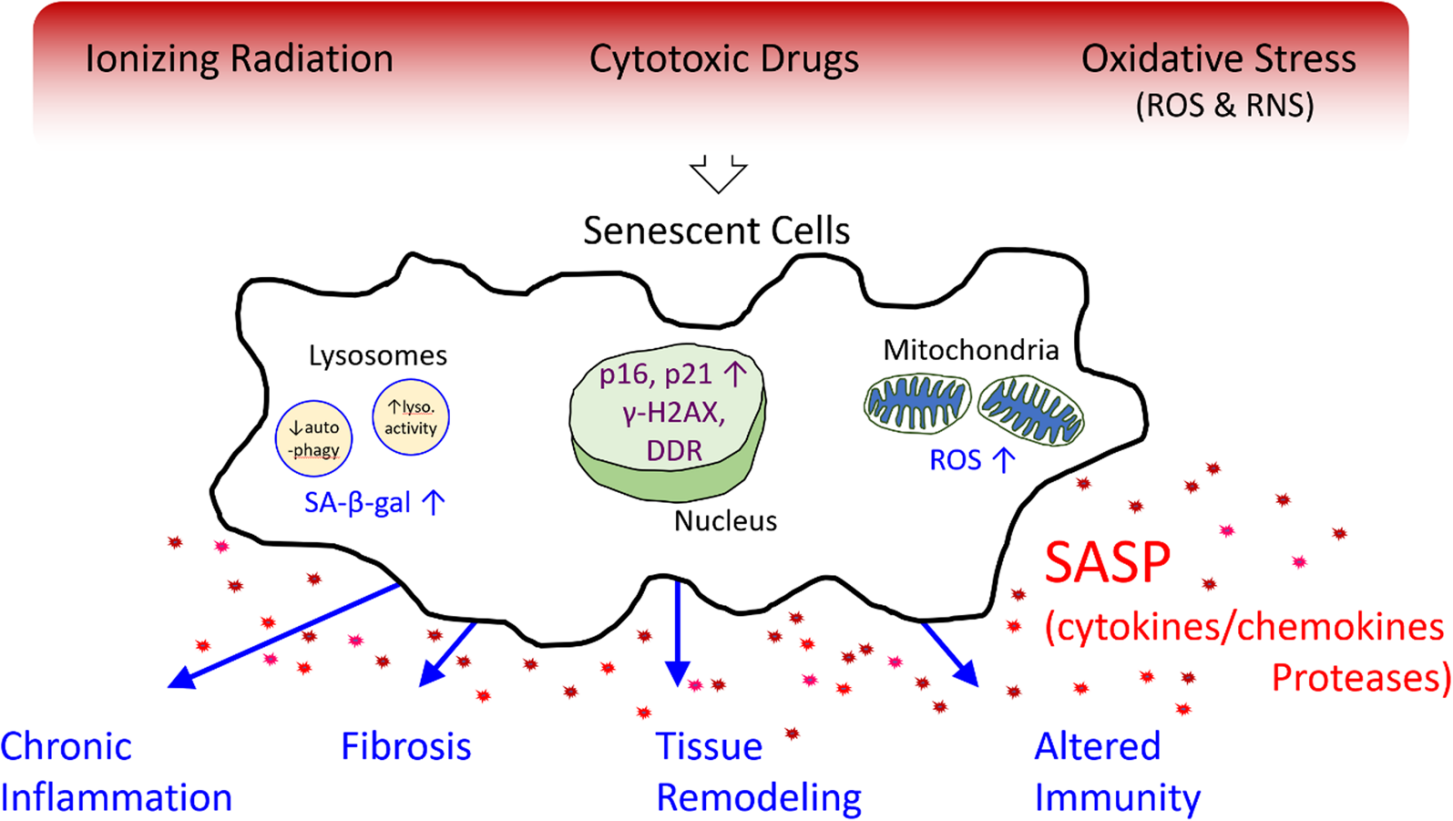
Senescence genesis, organelle specific molecular pathways consequences. Environmental stresses including ionizing radiation, cytotoxic agents and stress cause cells to express the senescent phenotype. Senescent cells are characterized by (1) increased lysosomal activity and decreased autophagy (2) expression of histone γ-H2AX (a marker of DNA strand breaks and telomere shortening), increased p16 and p21 (indicative of cell cycle arrest) and DDR (DNA damage response, an evolutionarily conserved signaling cascade, and (3) increased production of reactive oxygen species. These collectively promote a proinflammatory senescence associated secretory phenotype (SASP). The consequence of these processes increases chronic inflammation and fibrosis, promotes tissue remodeling and alters both innate and adaptive immunity

Cellular senescence, which is a normal consequence of aging, is characterized by irreversible cell cycle arrest in response to various stress stimuli, resistance to apoptosis and senescent-associated secretory phenotype (SASP). Cellular senescence is a cell fate decision and normal physiological event, which plays essential roles in development, prevention of cancer, and the wound healing process. However, when cells are subjected to sustained sub-lethal injury including radiation therapy or chemotherapy, continued oxidative stress and chronic inflammation prompt entry into cellular senescence. The chronic state of radiation-induced senescence together with secretion of pro-inflammatory factors, a phenomenon known as the SASP (see Fig. 1) contribute to the major pathology of radiation-induced normal tissue and organ injury. In this article, we systematically review research findings and highlight the contributions of senescent cells to the pathophysiology of radiation-induced normal tissue injury, as well as therapeutic options to eliminate radiation-induced senescence.

### Pathophysiology of radiation late effects

Classically, normal tissue injury following high doses of radiation is thought to result from either depletion of parenchymal and/or vascular endothelial cells. Attempts have been made over decades to determine whether parenchymal or endothelial progenitor cells are the primary targets responsible for the tissue damage; the debate continues. More recent molecular and cellular studies suggest that progressive damage to normal tissues after irradiation may be caused by radiation-induced long-lived free radicals resulting from reactive oxygen species (ROS) and reactive nitrogen species (RNS), and pro-inflammatory cytokines/chemokines, resulting in a deterioration of tissue and organ function [1–3]. Damaging ROS might arise from several sources including infiltrating activated leukocytes and macrophages. Further, other cells, such as fibroblasts, can be stimulated by pro-inflammatory cytokines to produce ROS. Tissue hypoxia resulting from vascular damage is another continual source of ROS generation [4]. The generation of these reactive molecules is part of the innate immune system and helps to rapidly clean the wound to reduce injury, but excessive production of ROS can lead to severe tissue damage including fibrosis and even neoplastic transformation. Strategies aimed at blocking effector molecules or otherwise reducing oxidative stress are attractive for preventing or mitigating radiation toxicity. For the last three decades or so, we and others have shown mitigating effects of a variety of agents including superoxide dismutase mimetics, statins, stem cell mobilizers and angiotensin converting enzyme inhibitors [1, 5–9]. In addition, pan-suppression of macrophage infiltration and cytokines/chemokines expression using a small molecule had a most impressive mitigating effect in normal tissues including skin and brain [5, 6].

An unmet critical question of normal tissue radiobiology is “*What is the source of chronic ROS and inflammation?”* The authors contend that one of the major sources of chronic inflammation is radiation-induced senescent cells.

### Biomarkers of radiation-induced senescence

**Fig. 2:**
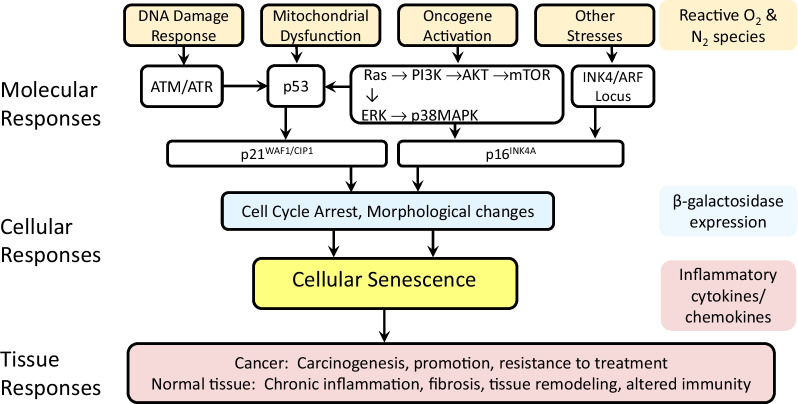
The four main molecular pathways governing cellular senescence: DNA Damage Response, Mitochondrial Dysfunction, Oncogene Activation and Other Stressors. The expression of p53 is a key regulator of cellular senescence; p53 is upregulated by ATM/ATR, mTOR and p38MAPK. In contrast, ARF in the Other Stressors pathway can inhibit p53 expression. Two other key regulators of cellular senescence are p21 and p16 both of which are measurable and two of the probable indicators of cellular senescence. Key consequences of cellular senescence are beta-galactosidase expression (useful as a biomarker of cellular senescence), inflammatory cytokines and chemokines, reactive oxygen and nitrogen species (which characterize the SASP phenotype)

Attempts have been made to classify molecular pathways involved in cellular senescence; using a modification of that proposed by Kumari and Jat [10], we propose four group: (1) the DNA Damage Response (DDR) pathway, (2) Mitochondrial Dysfunction, (3) Oncogene Activation and (4) Other Stresses. These are illustrated in Fig. 2. These molecular pathways have been implicated in aging of normal tissues (as described below) as well as cancer promotion and aggressiveness (also described below). Since radiation exposure is often used to simulate accelerated aging, it follows logically that the same four molecular pathways have a role in normal tissue injury following irradiation. The four molecular pathways are summarized in the following paragraphs.

### Biomarkers—DNA damage response pathway

DNA damage that results from proliferative exhaustion secondary to shortened telomeres or genotoxic stress either dependent or independent of reactive oxygen species (ROS) is orchestrated by the Ataxia Telangiectasia Mutated (ATM) and RAD3-related (ATR) kinases. ATM and ATR belong to the class-IV phosphoinositide 3-kinase (PI3K)-related kinase (PIKK) family and act as the sentries of genome stability; upon sensing DNA damage, they induce specific (i.e. G1/S G2/M and S-phase) cell cycle checkpoints through p53 increasing p21WAF1/CIP1 expression. p21WAF1/CIP1, also known as cyclin-dependent kinase inhibitor 1 or CDK-interacting protein 1, is a cyclin-dependent kinase inhibitor (CKI) that is capable of inhibiting all cyclin/CDK complexes [11], though is primarily associated with inhibition of CDK2 [12, 13]. Of primary importance to cellular senescence, p21WAF1/CIP1 binds to and inhibits the activity of CDK2–Cyclin E or CDK4/6–Cyclin D leading to cell arrest in G1/S.

### Biomarkers—mitochondrial dysfunction

Mitochondrial Dysfunction impacts similar cell cycle checkpoints through p53 expression, increasing p21WAF1/CIP1 and blocking cell cycle progression. Stresses such as low glucose, hypoxia, ischemia, heat shock and low NAD + /NADH cause low ATP in the cell and increased AMP-activated protein kinase (AMPK) expression. AMPK is a master regulator of cellular energy homeostasis. Persistent activation of AMPK leads to accelerated p53-dependent cellular senescence.

Oncogene-induced cellular senescence is a complex molecular program characterized by suppression of cell proliferation triggered in response to the aberrant activation of oncogenic signaling or the inactivation of a tumor-suppressor gene [14, 15]. For example, Ras, traditionally believed to promote unrestrained proliferation, has been implicated in oncogene-induced cellular senescence [16]. RAS is a GTPase that is frequently mutated in cancer and that affects a variety of cancer-driving processes [17]. RAS proteins, essential components of signalling pathways that emanate from cell surface receptors, lead to accelerated p53-dependent cellular senescence through both the Raf, p38MAPK pathway and the PI3K, AKT, mTOR pathway.

### Biomarkers—oncogene activation

The suppression of a cell death response by oncogenic RAS is a consequence of a perturbation of homeostatic balance between pro-apoptotic and anti-apoptotic signals. To keep up with the high energy needs of growing cells, the survival of RAS-transformed cells is further aided by metabolic reprogramming towards glycolysis that is mediated by MAPK- and PI3K-dependent regulation of hypoxia-inducible factor 1α (HIF-1α). Oncogenic RAS modulates the tumour microenvironment by promoting pro-angiogenic mechanisms and by altering host-mediated immune responses including HIF-mediated immune suppression. It is interesting to note that Song and colleagues propose that a combination of HIF-1α inhibitors with small molecules such as metformin and immunotherapy checkpoint blocking antibodies may boost anti-tumor immunity [18, 19] and enhance the anti-cancer effectiveness of high dose radiation therapy [20].

### Biomarkers—other stresses

Other stresses involve the INK4/ARF Locus to reduce p53-dependent cellular senescence through ARF inhibition of MDM2 and increase p16INK4A dependent cellular senescence [10, 21]. Hallmarks of senescence, in summary, are increased p21WAF1/CIP1 through p53-dependent/non-INK/ARF Locus pathways and p16INK4A through non-p53-dependent/INK/ARF Locus pathways [10]. Radiation can be expected to contribute to cellular senescence by all four molecular pathways: DNA Damage Response (DDR) pathway, (2) Mitochondrial Dysfunction, (3) Oncogene Activation and (4) Other Stresses.

### Biomarkers—available evidence and lack of consensus

It is a consensus among many senescence biologists that a universal marker of cellular senescence may never be found [22]. This is partly because of heterogeneity and diversity of tissues and their divergent responses to the plethora of genotoxic stimuli. The most common approach is to identify a panel of different markers based on cell cycle arrest (e.g. p16, p21), increased lysosomal compartment [e.g. senescence associated β-galactosidase (SA-β-gal)], structural changes associated with the DNA damage response (DDR; e.g. γH2AX) and additional traits specific for the SASP (e.g. increases in ROS, pro-inflammatory cytokines/chemokines, tissue proteases such as MMPs, etc.). Table 1 provides some common characteristics of cellular senescence that have the potential to be used as biomarkers of cellular senescence. Cell cycle exit is controlled by activation of the p53/p21 and/or p16/Rb tumor suppressor pathways. Unlike quiescent cells, senescent cells are non-responsive to mitogenic or growth factor stimuli. Consequently, increased expression of *CDKN1A* or *CDKN2A* RNA or the proteins they encode, p21 and p16Ink4a, respectively, are characteristic of senescent cells. However, these markers are not completely definitive because they may be induced in reversible cell cycle arrest or differentiation in specific cell types [23]. β-galactosidase activity, which is found in many normal cells under physiological conditions is significantly amplified in senescent cells as a result of increased lysosomal content [24]. Since SA-β-gal activity is detected in most senescent settings, both in vitro and in vivo, it is considered a de facto hallmark of senescence. However, SA-β-gal operates with a pH optimum of 6, in contrast to other lysosomal β-galactosidases (pH optimum 4–4.5), which necessitates careful controls that are not always reported [23]. Furthermore, some cells, notably hippocampal CA2 pyramidal and cerebellar purkinje neurons, express high endogenous levels of SA-β-gal, even at young ages [25], perhaps due to metabolic demands [26]. It is of note that components of the SASP have utility as confirmatory biomarkers of senescence, but are not stand-alone biomarkers, since most of them are not specific to senescence. Although a core SASP profile may exist, it has been recognized since the discovery of the SASP that its protein components can vary depending on cell type and inducing stimulus, as well as being temporally dynamic [27, 28; see below].

**Table 1:** Characteristics of cellular senescence impacting normal tissue response

Characteristics of senescence	Description/response
P16, p21	Cell cycle arrest at the expense of self-renewal or differentiation
Cell morphology	Cells become flat and larger
B-galactosidase	Cells appear blue with appropriate stain
Chronic ROS & RNS	Damages neighboring cells
Chronic inflammation	Chronic expression of cytokines/chemokines
Altered immunity	Increased dysfunction of both innate and adaptive immune responses
Aging	Fibrosis, tissue remodeling, impaired immune responses to pathogens and greater mortality and morbidity

Nevertheless, despite these difficulties, several studies document de novo expression of senescence-associated markers after therapeutic irradiation. Wang et al. reported [29] that total body irradiation selectively induced murine hematopoietic stem cell (HSC) senescence using two biomarkers, p16Ink4a and SA-β-gal. Of interest, the induction of HSC senescence was associated with a prolonged elevation of p21, p19ARF and p16Ink4a mRNA expression. In contrast, there were no changes in the biomarkers of irradiated hematopoietic progenitor cells [29]. Likewise, ionizing radiation induced endothelial senescence using the same biomarkers of senescence, SA-β-gal, p16Ink4a and p21 [30].

Radiation-induced pulmonary fibrosis (RIF) is one of the limiting factors in the treatment of advanced lung cancer using radiation therapy. Whether cellular senescence is responsible for RIF remains to be answered. Studies to date indicate that several cell types with biomarkers of senescence have been identified including SA-β-gal positive alveolar epithelial cells, putative alveolar stem cells, and mesenchymal stem cells [31–33].

Several investigators identified markers of cellular senescence 2–12 months after whole brain irradiation. Wong and co-workers [34] observed increased expression of the cell cycle- related regulators p16Ink4a and p19ARF in mouse hippocampus after 5 Gy, while Suman et al. [35] observed increased expression of p16Ink4a, p19ARF and p53 as well as indicators of oxidative damage in cerebral cortex after 1.6-2 Gy. Elevated cortical levels of RNA for *Cdkn1a* (p21), *Cdkn1b, Cdkn2a transcript 1* (p19ARF), *Cdkn2a* transcript 2 (p16Ink4A) were also reported [34, 36]. Irradiation induced senescence in mouse neural stem cells as indicated by increased expression of p16Ink4a, γH2AX, markers of reactive oxygen species and the SASP factor, Il-6 [23, 34, 37, 38]. Others showed the predominance of cellular senescence in astrocytes after radiotherapy for the malignant gliomas with increased expression of p16Ink4a and p21, as well as secretion of HGF and Il-6 as SASP factors [39]. Increased p53, but not telomere length, is an important mediator of astrocyte senescence [26]. Interestingly, the elimination of radiation-induced senescence in astrocytes using an inhibitor of Bcl-2 attenuated glioblastoma recurrence [36, 40, 41], while injecting irradiated, senescent human glioblastoma multiforme cells into an immunocompromised mouse resulted in faster tumor growth compared with non-irradiated, non-senescent cells [42].

Similar biomarkers of radiation-induced senescence in the mouse skin have been identified [43]. Many researchers have identified radiation-induced senescence in cultured cells from many organs in vitro [44–47], and even from transformed cell lines [24].

### Senescence associated secretory phenotype (SASP)

SASP is a phenotype associated with senescent cells wherein those cells secrete a complex mixture containing hundreds of proteins, including pro-inflammatory cytokines/chemokines, immune modulators, tissue-damaging proteases, factors that can adversely affect stem and progenitor cell function, homeostatic factors, ceramides, bradykinins and growth factors [48–51]. Senescent cells also release exosomes and ectosomes such as enzymes, microRNA, DNA fragments, and the anti-apoptotic protein, Bcl-xL. Although the early phase of SASP has biologically beneficial effects in wound healing and tissue remodeling, SASP is the primary cause of the detrimental chronic effects of senescent cells. Senescence does not only affects events inside the cell but has the potential to affect the surroundings through paracrine loops and/or entry into circulation [28]. The SASP is heterogenous, although the full extent of the heterogeneity is only starting to be explored. Transcriptomic analyses of senescent cells [52–57] assume that gene expression changes will be predictive of SASP constituents. SASP proteomic atlases are also starting to be generated [46]. Cell type appears to be the most significant factor in affecting SASP constituent heterogeneity [53, 55]. However, inducing stimulus is also important and has led some investigators to begin grouping SASP factors into functional categories, with unique acronyms. A group of specific SASP factors regulated by Nfkb signaling (NASP), p53 associated factors (PASP) and Stat3 regulated factors have been identified [58, 59]. For example, viral vector driven over-expression of p16 [60] or pharmacological treatment with CDK4/6 inhibitors [59] fail to induce Nfkb regulated SASP factors such as IL6, but rather induce RNAs encoding factors associated with p53 including Igfbp3, Lif, and Tollip. Finally, SASP constituents vary over time [51]. Thus, definitive compendia of SASP components will require additional investigation.

Senescent cells are highly metabolically active, producing large amounts of above mentioned SASP factors, which is why senescent cells consisting of only 2–3% of tissue cells can be a major cause of aging associated diseases [61, 62]. Given that humans contain an estimated 37 × 10^12^ cells, including 1 × 10^6^ pituitary cells, the small fraction of senescent cells outnumber professional secretory cells [63] to produce widespread systemic effects, including within the immune system. SASP factors such as IL-6 and TNFα enhance T-cell apoptosis, thereby impairing the capacity of the adaptive immune system [64]. Chronic inflammation due to SASP can also suppress immune system function. Immune system responses to senescent cells and senolytics have been reviewed recently [65].

The SASP is regulated at multiple levels, including transcription, translation, mRNA stability and secretion. One of the important regulatory pathways is mammalian target of rapamycin (mTOR). Interleukin1-alpha is found on the surface of senescent cells, where it contributes to the production of SASP. mTOR inhibition prevents the IL-1α from degrading transcripts of numerous components of SASP factors [66, 67]. The use of mTOR inhibitors showed senostatic effect in various animal studies [68, 69; see below].

### The role of radiation-induced senescence in tumor tissue

While most research on cellular senescence has been performed on non-cancerous cells, however, cancer cells can be equally induced to cellular senescence through a variety of stress and damage signals including radiation and cytotoxic chemotherapy. A prime first responders in the DNA damage response, non-homologous end-joining and homologous recombination, are two main pathways for repairing double strand breaks, which are potent stimuli for inducing cellular senescence. Senescent cells exhibit apoptosis resistance, metabolic activity and secretion of pro-inflammatory and proliferative molecules (SASP). The effect of the SASP is highly dependent on context and cell type and variable during the different stages of cancer progression [70, 71]. Factors influencing the role of cellular senescence in the tumor tissue widely vary in part due to the tumor tissue heterogeneity, the oncogenic status, immune cell recognition by acute vs chronic senescence and radiation dose regimen, to name a few [72–74]. For example, acute induction of cellular senescence is considered important for cancer prevention by stimulating the immune system to rapidly eliminate the genetically unstable cells, while chronic cellular senescence due to persistent stress signals (ROS, chronic inflammation) and the accumulation of dysfunctional senescent cells is unable to remove by immune cells, whereas chronic cellular senescence creates a tumor promoting environment through a secretion of SASP including IL-1 alpha/beta, IL-6/8, MMPs, VEGF, TGF-beta, HFH, etc. The tumor microenvironment stimulates tumor cell proliferation, angiogenesis and epithelial to mesenchymal transition. Foregoing factors contribute to the increase in the tumor radioresistance. Chronic cellular senescence also contribute to the radiation-induced late effects in the normal tissues and organs such as lung and skin fibrosis, cognitive dysfunction/necrosis to name a few. Overall, the SASP of senescent cancer cells is considered to be primarily detrimental in therapy resistance, immunosuppression and metastasis [70, 75].

It is well established that the efficacy of tumor radiotherapy depends on the total dose of radiation, dose per fraction and duration of fractionation regimen. Usual radiation fraction size in the clinical radiotherapy ranges from 1.8 to 2.5 Gy per fraction. Most tumor and normal cells sustain sub-lethal injury which would result in the cellular senescence, but a fraction of tumor cells undergo lethal cell death through either apoptosis or mitotic catastrophe as shown in the Fig. 1. When a radiation dose increases above 10 Gy per fraction, most cells will sustain lethal irreparable damage while a fraction of tumor cells undergoes cellular senescence [76]. It is reasonably well established that the radiosurgery/stereotactic body radiotherapy (SBRT) has consistently shown a superior tumor control rate relative to the conventional fractionated radiotherapy [77, 78]. Although the initial introduction of SBRT is aimed to exploit the superior geometrical distribution of radiation dose to the small target tumor tissue relative to the surrounding normal tissue, there is mounting evidence that additional radiobiological factors would contribute to the increase in the tumor control rate perhaps including vascular and immune effect of SBRT [79, 80]. We posit that the radiation-induced cellular senescence may also play important contributing factor in the increase tumor control rate. Since the quantity of radiation-induced cellular senescence in the normal tissue in the conventional RT (usually 20–30%) vs SBRT (less than 5%) is disproportionally high, the tumor recurrence rate and normal tissue damage would be expected to be high in the conventional fractionated RT relative to SBRT in part to the detrimental effect of SASP from cellular senescence as discussed in the foregoing section. Indeed, a recent paper shows the elimination of senescent astrocytes induced by radiation reduces the tumor recurrence of the radioresistant malignant glioma in the brain [40].

## Therapeutic opportunities

### Senolytics and senostatics

**Fig. 3:**
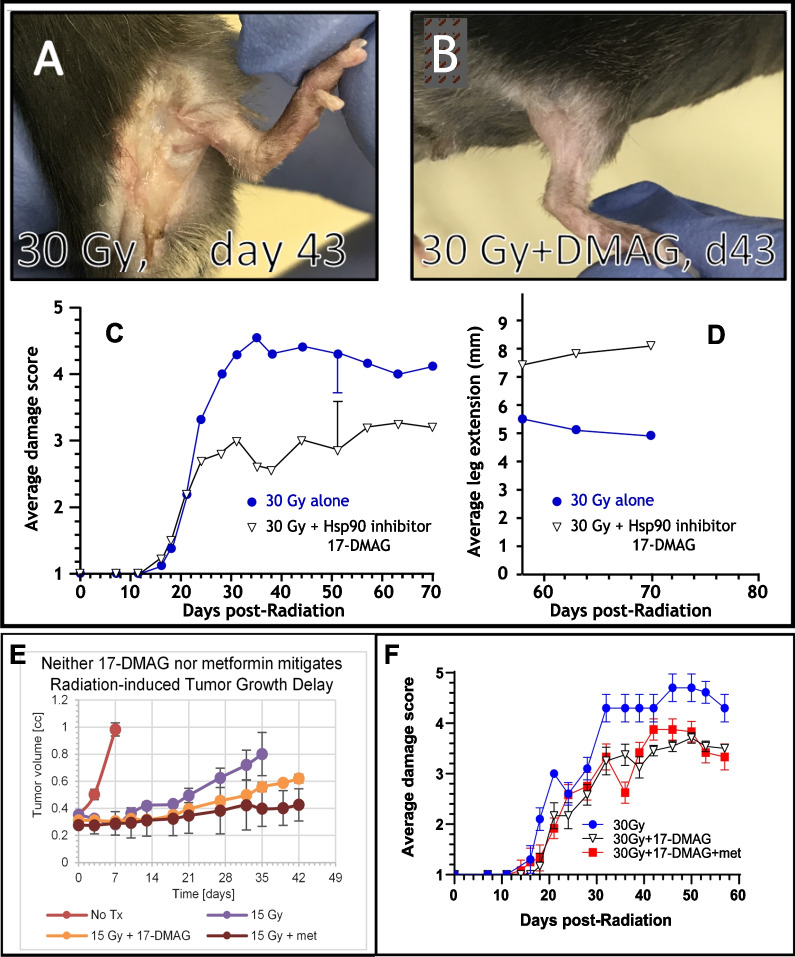
Radiation skin injury is mitigated by the senolytic agent, 17-DMAG. Seven days after 30 Gy radiation (day 0), treatment with 17-DMAG was initiated. Panel **A**: Typical skin damage in a mouse six weeks after 30 Gy radiation alone. Panel **B**: Typical skin damage to a mouse at the same time point after 30 Gy plus 17-DMAG. Panel C: The time course of skin damage following 30 Gy alone or 30 Gy plus 17-DMAG using a semi-quantitative scale, and, Panel **D**, the corresponding leg contraction. Curves were statistically different; as an example, standard deviations illustrated at day 50 show a statistically significant separation between groups. Skin injury semi-quantitative scale is 1 = normal, 2 = erythema, 3 = dry desquamation, 4 = moist desquamation and 5 = necrosis. Typically, scores of 3 and under resolve with time whereas scores greater than 3 do not. Each curve is from 5 mice. Error bars shown represent standard deviation. Panels **E** and **F** show the effect of a senolytic agent and senostatic agent to increase the therapeutic gain; each strategy elicits an anti-cancer effect and the combined administration mitigates normal tissue radiation injury. Panel **E** illustrates an increased A-549 tumor growth delay following administration of either a senolytic agent, 17-DMAG or a senostatic agent, metformin. At day 35, tumor volumes following 15 Gy + metformin was statistically different from that of 15 Gy alone (other groups did not reach significance and only trends were observed). Panel **F** shows that combining a senolytic and senostatic agent mitigates radiation-induced skin injury in C57BL/6 mice (data from a separate experiment as that shown in **A**–**D**). Note that combining the senostatic (metformin) with a senolytic (17-DMAG) did not abrogate the mitigation of radiation injury. At day 50, average damage score of mice receiving either 30 Gy + 17-DMAG or 30 Gy + 17-DMAG + metformin were statistically different from that of mice receiving 30 Gy radiation alone (although adding metformin did not improve average damage score). Each data point represents at least 10 mice for tumor growth delay and at least 5 mice for skin damage study. Error bars shown represent the standard deviation

Senolytics are a class of drugs that selectively eliminate senescent cells. Multiple pharmacological strategies are under investigation to remove senescent cells. They include small molecules, peptides, and antibodies [81–86]. Senescent cells are generally resistant to apoptosis. Some senolytic agents are cell and tissue specific; others are not. To date, five or six different signaling pathways have been identified and targeted drugs are being developed. These include Bcl-2, PI3K/AKT/mTOR, HIF-alpha pathways, TK inhibitors and HSP-90 inhibitors, to name a few [83]. Examples of senolytics include Dasatinib, Quercetin, Fisetin, and Navitoclax [82]. However, the current generation of senolytics targeting these proteins have some limitations in terms of safety, specificity and broad-spectrum activity. It is interesting to note that most senolytic drugs were initially being developed as anti-cancer agents, the so-called targeted cancer drugs, since some of the signaling pathways in tumor and senescent cells overlap each other. Our new preliminary data show the potential of senolytic as well as anti-cancer agents to illustrate the foregoing point. Alvespimycin (17-DMAG), an HSP-90 inhibitor, reduced normal tissue damage after a radiation exposure without compromising radiotherapy effectiveness [87]. The mitigating effect of 17-DMAG alone on acute skin damage and late effects in response to a single dose of 30 Gy exposure is shown in Fig. 3. Using another class of senolytics, Kirkland and his team have shown some functional and structural improvement in cardiovascular function, and radiation-induced muscle weakness using the combined senolytics, dasanitib and quercetin [82]. In a small Phase I clinical study without a placebo control, the dasanitib and quercetin combination appeared to be well tolerated and to alleviate frailty in elderly men and women with a serious lung disease. Other early data on the effectiveness in humans have been mixed, although 10 additional open label trials are ongoing, including one in HSC transplant survivors (clinicaltrials.gov). Using another class of senolytics, navitoclax, a Bcl-2 family inhibitors, improved radiation-induced pulmonary fibrosis [88], radiation-induced hematotoxicity, age related HSC dysfunction [89] and delayed malignant glioma recurrence by eliminating the radiation-induced senescent astrocytes [40]. The potential of navitoclax to mitigate normal tissue radiation damage while sensitizing radiation cytotoxicity in tumors is further supported by navitoclax’s ability to overcome hypoxia-driven radiosensitivity [90]. Although navitoclax is an FDA approved drug for the treatment of chronic lymphocytic leukemia, the main dose limiting toxicity has been thrombocytopenia. As with many other current cancer therapeutics, the most likely scenario of using senolytics would be utilizing a combinatorial approach.

In contrast to senolytics, senostatics do not kill senescent cells but inhibit paracrine signaling and thus limit the spread of senescence via the so-called bystander effect. Antioxidants including multiple flavonoids, polyphenols and other phyto-chemicals may have a senostatic effect. mTOR pathway inhibitors and mitochondrial function (complex I) have significant senostatic potential [91, 92]. Metformin and Rapamycin are examples of senostatic agents [84]. Unlike senolytics targeting a specific signaling pathway, senostatics target not only senescent cells but also non-senescent related functions. Nevertheless, a short-term treatment of mice with rapamycin, metformin (an anti-diabetic drug), or dietary restriction decreased frequencies of cells positive for multiple senescence markers [93–95]. Rapamycin appeared to mimic the effects of calorie restriction and induced autophagy (a process the decline of which is associated with a number of age-related diseases). A clinical trial of anti-aging in humans is being planned using metformin at 1500 mg per day, according to the American Federation for Aging Research. Many questions remain to be addressed before launching large scale human trials using either senolytics or senostatics or both. These include the dosage, timing and duration of treatment (i.e. intermittent vs continuous); further, endpoints for evaluation such as monitoring bio-markers of senescence and functionality of the therapy efficacy need to be addressed.

An area of future study is to test whether combining senolytics and senostatics has the potential to increase tumor control and simultaneously reduce normal tissue injury induced by radiation. It is of note that metformin in the context of an inhibitor of NF-κB improved cancer cytotoxicity in vitro and in vivo by interfering with senescence-associated cytokine production [96]. Figure 3E illustrates the potential benefit of combining 17-DMAG with metformin. Metformin alone in mice resulted in mitigation of radiation injury to the same extent as did the senolytic, 17-DMAG, in the same animal model of skin and muscle injury (data not shown). Interestingly, combining the senolytics and senostatic in this model did not further reduce radiation damage; one interpretation is that the target for senolytic and senostatic mitigation of tissue injury is the same (Fig. 3F).

### Cellular reprogramming

Another therapeutic option to eliminate or reverse cellular senescence comes from cellular reprogramming approaches. Expressing so called Yamanaka factors, OCT4, SOX2, KLF4 and c-MYC (OSKM) converts somatic cells into induced pluripotent stem cells (iPSCs). Ocampo et al. have shown the potential of partial reprogramming in tackling aging [97–99]. Unlike previous studies that used Yamanaka factors in vivo which could initiate cancer development or teratoma formation, Ocampo and his co-workers have successfully demonstrated that tumor formation can be avoided by short-term induction of OSKM. Further, cyclic induction of OSKM in vivo ameliorated hallmarks of aging and improved the regenerative capacity of pancreas and muscle following injury in physiologically aged mice. More recently, Sarkar, Rando et al. described a feasible way to deliver Yamanaka factors to cells taken from patients with osteoarthritis by dosing cells kept in cultures with small doses of the factors [100]. The result showed not only restoration of lost functionality in diseased cells and aged stem cells but also preservation of cellular identity. Also, it is interesting to note that they used a non-integrative, mRNAs-based platform of transient cellular reprogramming. In vivo transient expression of nuclear reprogramming factors holds great promise for reversal of senescence and tissue repair and regeneration. Reprogramming cells in vivo has been shown to be possible with recent clinical successes employing CRISPR technology (e.g., in patients with genetic diseases such as sickle cell anemia) [101, 102].

## Conclusion

There is mounting evidence showing that radiation-induced senescence in both tumor and normal tissues contributes to tumor recurrence, metastasis, and resistance to therapy while senescent cells in the normal tissue and organ are a source of many late damaging effects. The authors propose the hypothesis that the source of chronic ROS and inflammation is radiation-induced senescent cells; this has not been confirmed and is an area of active research that may lead to a new therapeutic option. Advances in the cellular and molecular pathways of cellular senescence provide novel strategies to enhance therapeutic ratio of radiation therapy. Pre-clinical data on the radiation-induced senescence and late tissue damage using senolytics and senostatics provide a promising avenue for radiotherapy research.

## Data Availability

All raw data is available upon request to the corresponding author (SB).

## References

[1] Kim JH, Jenrow KA, Brown SL (2014). Mechanisms of radiation-induced normal tissue toxicity and implications for future clinical trials. Radiat Oncol J.

[2] Giaccia AJ (2014). Molecular radiobiology: the state of the art. J Clin Oncol.

[3] Citrin DE, Mitchell JB (2017). Mechanisms of normal tissue injury from irradiation. Semin Radiat Oncol.

[4] Zhao W, Diz DI, Robbins ME (2007). Oxidative damage pathways in relation to normal tissue injury. Br J Radiol.

[5] Jenrow KA, Brown SL, Kolozsvary AJ, Lapanowski K, Kim JH (2014). Time-dependent inhibition of pan-inflammatory cytokines mitigates radiation-induced skin injury in mice. Radiat Res.

[6] Jenrow KA, Brown SL, Lapanowski K (2013). Selective inhibition of microglia-mediated neuroinflammation mitigates radiation-induced cognitive impairment. Radiat Res.

[7] Kim JH, Brown SL, Kolozsvary A (2004). Modification of radiation injury by ramipril, inhibitor of angiotensin-converting enzyme, on optic neuropathy in the rat. Radiation Res.

[8] Kim JH, Kolozsvary A, Jenrow KA (2012). Plerixafor, a CXCR4 antagonist, mitigates skin radiation-induced injury in mice. Radiat Res.

[9] Yan S, Brown SL, Kolozsvary A (2008). Mitigation of radiation-induced skin injury by AAV2-mediated MnSOD gene therapy. J Gene Med.

[10] Kumari R, Jat P (2021). Mechanisms of cellular senescence: cell cycle arrest and senescence associated secretory phenotype. Front Cell Dev Biol.

[11] Xiong Y, Hannon GJ, Zhang H, Casso D, Kobayashi R, Beach D (1993). p21 is a universal inhibitor of cyclin kinases. Nature.

[12] Abbas T, Dutta A (2009). p21 in cancer: intricate networks and multiple activities. Nat Rev Cancer.

[13] Harper JW, Adami GR, Wei N, Keyomarsi K, Elledge SJ (1993). The p21 Cdk-interacting protein Cip1 is a potent inhibitor of G1 cyclin-dependent kinases. Cell.

[14] Liu XL, Ding J, Meng LH (2018). Oncogene-induced senescence: a double edged sword in cancer. Acta Pharmacol Sin.

[15] Chandeck C, Mooi WJ (2010). Oncogene-induced cellular senescence. Adv Anat Pathol.

[16] Dimauro T, David G (2010). Ras-induced senescence and its physiological relevance in cancer. Curr Cancer Drug Targets.

[17] Pylayeva-Gupta Y, Grabocka E, Bar-Sagi D (2011). RAS oncogenes: weaving a tumorigenic web. Nat Rev Cancer.

[18] Ma R, Yi B, Riker AI, Xi Y (2020). Metformin and cancer immunity. Acta Pharmacol Sin.

[19] Shao S, Zhao L, An G, Zhang L, Jing X, Luo M, Li W, Meng D, Ning Q, Zhao X (2020). Metformin suppresses HIF-1α expression in cancer-associated fibroblasts to prevent tumor-stromal cross talk in breast cancer. FASEB J.

[20] Song CW, Kim H, Cho H, Kim MS, Paek SH, Park HJ, Griffin RJ, Terezakis S, Cho LC (2022). HIF-1α inhibition improves anti-tumor immunity and promotes the efficacy of stereotactic ablative radiotherapy (SABR). Cancers (Basel).

[21] Sherr CJ (2012). Ink4-Arf locus in cancer and aging. Wiley Interdiscip Rev Dev Biol.

[22] Gonzalez-Gualda E, Baker AG, Fruk L (2021). A guide to assessing cellular senescence in vitro and in vivo. FEBS.

[23] Gillispie GJ, Sah E, Krishnamurthy S, Ahmidouch MY, Zhang B, Orr ME (2021). Evidence of the cellular senescence stress response in mitotically active brain cells-Implications for cancer and neurodegeneration. Life (Basel).

[24] Lee BY, Han JA, Im JS (2006). Senescence-associated beta-galactosidase is lysosomal beta-galactosidase. Aging Cell.

[25] Piechota M, Sunderland P, Wysocka A (2016). Is senescence-associated beta-galactosidase a marker of neuronal senescence?. Oncotarget.

[26] Sah E, Krishnamurthy S, Ahmidouch MY, Gillispie GJ, Milligan C, Orr ME (2021). The cellular senescence stress response in post-mitotic brain cells: cell survival at the expense of tissue degeneration. Life (Basel).

[27] van Deursen JM (2014). The role of senescent cells in ageing. Nature.

[28] Basisty N, Kale A, Patel S, Campisi J, Schilling B (2020). The power of proteomics to monitor senescence-associated secretory phenotypes and beyond: toward clinical applications. Expert Rev Proteom.

[29] Wang Y, Schulte BA, LaRue AC (2006). Total body irradiation selectively induces murine hematopoetic stem cell senescence. Blood.

[30] Wang Y, Boerma M, Zhou D (2016). Ionizing radiation-induced endothelial cell senescence and cardiovascular diseases. Radiat Res.

[31] Epperly MW, Shields D, Fisher R (2021). Radiation-induced senescence in p16+/LUC mouse lung compared to bone marrow multilineage hematopoetic progenitor cells. Radiat Res.

[32] He Y, Thummuri D, Zheng G (2019). Cellular senescence and radiation-induced pulmonary fibrosis. Transl Res.

[33] Mukherjee A, Epperly MW, Shields D (2021). Ionizing irradiation=induced Fgr in senescent mediates fibrosis. Cell Death Discov..

[34] Cheng Z, Zheng YZ, Li Y (2017). Cellular senescence in mouse hippocampus after irradiation and the role of p53 and p21. J Neuropathol Exp Neurol.

[35] Suman S, Rodriguez OC, Winters TA, Fornace AJ, Albanese C, Datta K (2013). Therapeutic and space radiation exposure of mouse brain causes impaired DNA repair response and premature senescence by chronic oxidant production. Aging (Albany NY).

[36] Yabluchanskiy A, Tarantini S, Balasubramanian P (2020). Pharmacological or genetic depletion of senescent astrocytes prevents whole brain irradiation-induced impairment of neurovascular coupling responses protecting cognitive function in mice. Geroscience.

[37] Ungvari Z, Podlutsky A, Sosnowska D (2013). Ionizing radiation promotes the acquisition of a senescence-associated secretory phenotype and impairs angiogenic capacity in cerebromicrovascular endothelial cells: role of increased DNA damage and decreased DNA repair capacity in micro- vascular radiosensitivity. J Gerontol a Biol Sci Med Sci.

[38] Li T, Li L, Li F, Liu Y (2015). X-ray irradiation accelerates senescence in hippocampal neural stem/progenitor cells via caspase-1 activation. Neurosci Lett.

[39] Turnquist C, Beck JA, Horikawa I (2019). Radiation-induced astrocyte senescence is rescued by delta133p53. Neuro-Oncol.

[40] Fletcher-Sananikone E, Kanji S, Tonimastu N (2021). Elimination of radiation-induced senescence in the brain tumor microenvironment attenuates glioblastoma recurrence. Cancer Res.

[41] Prasanna PG, Citrin DE, Hildesheim J (2021). Therapy-induced senescence: opportunities to improve anti-cancer therapy. J Natl Cancer Inst.

[42] Jeon HY, Kim JK, Ham SW, Oh SY, Kim J, Park JB, Lee JY, Kim SC, Kim H (2016). Irradiation induces glioblastoma cell senescence and senescence-associated secretory phenotype. Tumour Biol.

[43] McCart EA, Thangapazham RL, Lombardini ED (2017). Accelerated senescence in skin in a murine model of radiation-induced multi-organ injury. J Radiat Res.

[44] Zou Y, Zhang N, Ellerby LM, Davalos AR, Zeng X, Campisi J, Desprez PY (2012). Responses of human embryonic stem cells and their differentiated progeny to ionizing radiation. Biochem Biophys Res Commun.

[45] Schneider L, Pellegatta S, Favaro R, Pisati F, Roncaglia P, Testa G, Nicolis SK, Finocchiaro G, Adda di Fagagna F (2013). DNA damage in mammalian neural stem cells leads to astrocytic differentiation mediated by BMP2 signaling through JAK-STAT. Stem Cell Rep.

[46] Basisty N, Kale A, Jeon OH (2020). A proteomic atlas of senescence-associated secretomes for aging biomarker development. PLoS Biol.

[47] Zhu Y, Prata LGPL, Gerdes EOW (2022). Orally-active, clinically-translatable senolytics restore α-Klotho in mice and humans. EBioMed.

[48] Coppe JP, Kauser K, Campisi J (2006). Secretion of vascular endothelial growth factor by primary human fibroblasts at senescence. J Biol Chem.

[49] Coppe JP, Desprez PY, Krtolica A (2010). The senescence associated secretory phenotype: the dark side of tumor suppression. Annu Rev Pathol.

[50] Kumari R, Parmjit J (2021). Mechanisms of cellular senescence: cell cycle arrest and senescence associated secretory phenotype. Front Cell Dev Biol.

[51] Birch J, Gil J (2020). Senescence and the SASP: many therapeutic avenues. Genes Dev.

[52] Zhang H, Pan KH, Cohen SN (2003). Senescence-specific gene expression fingerprints reveal cell-type-dependent physical clustering of up-regulated chromosomal loci. Proc Natl Acad Sci USA.

[53] Hernandez-Segura A, de Jong TV, Melov S, Guryev V, Campisi J, Demaria M (2017). Unmasking transcriptional heterogeneity in senescent cells. Curr Biol.

[54] Uyar B, Palmer D, Kowald A (2020). Single-cell analyses of aging, inflammation and senescence. Ageing Res Rev.

[55] Jochems F, Thijssen B, De Conti G (2021). The cancer SENESCopedia: a delineation of cancer cell senescence. Cell Rep.

[56] Kiss T, Nyul-Toth A, DelFavero J (2022). Spatial transcriptomic analysis reveals inflammatory foci defined by senescent cells in the white matter, hippocampi and cortical grey matter in the aged mouse brain. Geroscience.

[57] Xu P, Wang M, Song WM (2022). The landscape of human tissue and cell type specific expression and co-regulation of senescence genes. Mol Neurodegener.

[58] Rao SG, Jackson JG (2016). SASP: tumor suppressor or promoter? Yes!. Trends Cancer.

[59] Wang B, Brandenburg S, Hernandez-Segura A (2020). Pharmacological CDK4/6 inhibition unravels a p53-induced secretory phenotype in senescent cells. Biorxiv.

[60] Coppe JP, Rodier F, Patil CK, Freund A, Desprez PY, Campisi J (2011). Tumor suppressor and aging biomarker p16(INK4a) induces cellular senescence without the associated inflammatory secretory phenotype. J Biol Chem.

[61] Childs BG, Gluscevic M, Baker DJ (2017). Senescent cells: an emerging target for diseases of ageing. Nat Rev Drug Discov.

[62] Francesi C, Campisi J (2014). Chronic inflammation and its potential contribution to age-associated diseases. J Geront.

[63] Lopes-Paciencia S, Saint-Germain E, Rowell MC, Ruiz AF, Kalegari P, Ferbeyre G (2019). The senescence-associated secretory phenotype and its regulation. Cytokine.

[64] Bartleson JM, Radenkovic D, Verdin E (2021). SARS-CoV-2, COVID-19 and the ageing immune system. Nature Aging.

[65] Chibaya L, Snyder J, Ruscetti M (2022). Senescence and the tumor-immune landscape: Implications for cancer immunotherapy. Semin Cancer Biol.

[66] Biran A, Zada L, Karam PA (2017). Quantitative identification of senescent cells in aging and disease. Aging Cell.

[67] Herranz N, Gil J (2018). Mechanisms and functions of cellular senescence. J Clin Invest.

[68] Papadopoli D, Boulay K, Kazak L (2019). mTOR as a central regulator of lifespan and aging. F1000Research.

[69] Kaeberlin M, Galvan V (2019). Rapamycin and Alzheimer's disease: time for a clinical trial. Sci Transl Med.

[70] Wang L, Lankhorst L, Bernards R (2022). Exploiting senescence for the treatment of cancer. Nat Rev Cancer.

[71] Chen Z, Cao K, Xia Y (2019). Cellular senescence in ionizing radiation. Oncol Rep.

[72] He Y, Thummuri D, Zheng G (2019). Cellular senescence and radiation-induced fibrosis. Transl Res.

[73] Milanovic M, Fan DNY, Belenki D (2018). Senescence-associated reprogramming promotes cancer stemness. Nature.

[74] Wyld L, Bellantuono I, Tchkonia T (2020). Senescence and cancer: a review of clinical implications of senescence and senotherapies. Cancers (Basel).

[75] Patel NH, Sohal SS, Manjili MH (2020). The roles of autophagy and senescence in the tumor cell response to radiation. Radiat Res.

[76] Kim BC, Yoo HJ, Lee HC (2014). Evaluation of premature senescence and senescence biomarkers in carcinoma cells and xenograft mice exposed to single or fractionated irradiation. Oncol Rep.

[77] Citrin DE (2017). Recent developments in radiotherapy. N Engl J Med.

[78] Bijlani A, Aguzzi G, Schaal DW (2013). Stereotactic radiosurgery and stereotactic body radiation therapy cost-effectiveness results. Front Oncol.

[79] Brown SL, Nagaraja TN, Aryal MP (2015). MRI-tracked tumor vascular changes in hours after single-fraction irradiation. Radiat Res.

[80] Song CW, Terezakis S, Emami B (2020). Indirect cell death and the LQ model in SBRT and SRS. J Radiosurg SBRT.

[81] Kirkland JL, Tchkonia T (2017). Cellular senescence: a translational perspective. EBioMedicine.

[82] Kirkland JL, Tchkonia T (2020). Senolytic drugs: from discovery to translation. J Intern Med.

[83] Fuhrmann-Stroissnigg H, Ling YY, Zhao J (2017). Identification of HSP90 inhibitors as a novel class of senolytics. Nat Commun.

[84] Short S, Fielder E, Miwa S (2019). Senolytics and senostatics as adjuvant therapy. EBioMedicine.

[85] Kang C (2019). Senolytics and senostatics: A two-pronged approach to target cellular senescence for delaying aging and age-related diseases. Mol Cells.

[86] Zhu Y, Tchkonia T, Pirtsh A (2015). The achilles heel of senescent cells: from transcriptome to senolytics drugs. Aging Cell.

[87] Kim JH, Kolozsvary A, Brown SL. Senolytics mitigate radiation-induced normal tissue damage. In: 64th radiation res annual meeting; 2018 September 23–26; Chicago, IL, USA.

[88] Pan J, Li D, Xu Y (2017). Inhibition of Bcl-2/xL with ABT-263 selectively kills senescent type II pneumocites and reverses persistent pulmonary fibrosis induced by ionizing radiation in mice. Int J Radiat Oncol Biol Phys.

[89] Chang J, Wang L, Shao L (2016). Clearance of senescent cells by ABT263 rejuvenates aged hematopoetic cells in mice. Nat Med.

[90] Ritter V, Krautter F, Klein D, et al. Bcl-2/Bcl-xL inhibitor ABT-263 overcomes hypoxia-driven radioresistence and improves radiotherapy. Cell Death Dis. 2021;12(7):694. Erratum in: Cell Death Dis. 2022;13(4):367. 10.1038/s41419-021-03971-7 PMC8277842 34257274

[91] Janubova M, Zitnanova I (2017). Effects of bioactive compounds on senescence and components of senescence associated secretory phenotypes in vitro. Food Funct.

[92] Nelson G, Kucheryavenko O, Wordsworth J (2018). The senescent bystander effect is caused by ROS-activated NF-kappaB signaling. Mech Ageing Dev.

[93] Moiseeva O, Deschênes-Simard X, St-Germain E (2013). Metformin inhibits the senescence-associated secretory phenotype by interfering with IKK/NF-kappaB activation. Aging Cell.

[94] Brown SL, Kolozsvary A, Isrow DM (2019). A novel mechanism of high dose radiation sensization by metformin. Front Oncol.

[95] Yi G, He Z, Zhou X (2013). Low concentration of metformin induces a p53-dependent senescence in hepatoma cells via activation of the AMPK pathway. Int J Oncol.

[96] Schoetz U, Klein D, Hess J (2021). Early senescence and production of senescence-associated cytokines are major determinants of radioresistance in head-and-neck squamous cell carcinoma. Cell Death Dis.

[97] Ocampo A, Reddy P, Martinez-Redondo P (2016). In vivo amelioration of age-associated hallmarks by partial reprogramming. Cell.

[98] Alle Q, Le Borgne E, Milhavet O (2021). Reprogramming: emerging strategies to rejuvenate aging cells and tissues. Mol Sci.

[99] De Lima Camil L, Quinla RBA (2021). A ride through the epigenetic landscape: aging reversal by reprogramming. GeroSci.

[100] Sarkar TJ, Quarta M, Mukherjee S (2020). Reprogramming factors promotes multifaceted amelioration of aging in human cells. Nat Commun.

[101] Kan MJ, Doudna JA (2022). Treatment of genetic diseases with CRISPR genome editing. JAMA.

[102] Uddin F, Rudin CM, Sen T (2020). CRISPR gene therapy: applications, limitations, and implications for the future. Front Oncol.

